# Costs and benefits of voluntary attention in crows

**DOI:** 10.1098/rsos.230517

**Published:** 2023-08-16

**Authors:** Linus Hahner, Andreas Nieder

**Affiliations:** Animal Physiology Unit, Institute of Neurobiology, University of Tübingen, 72076 Tübingen, Germany

**Keywords:** crow, executive functions, attention, volition

## Abstract

Behavioural signatures of voluntary, endogenous selective attention have been found in both mammals and birds, but the relationship between performance benefits at attended and costs at unattended locations remains unclear. We trained two carrion crows (*Corvus corone*) on a Posner-like spatial cueing task with dissociated cue and target locations, using both highly predictive and neutral central cues to compare reaction time (RT) and detection accuracy for validly, invalidly and neutrally cued targets. We found robust RT effects of predictive cueing at varying stimulus-onset asynchronies (SOA) that resulted from both advantages at cued locations and costs at un-cued locations. Both crows showed cueing effects around 15–25 ms with an early onset at 100 ms SOA, comparable to macaques. Our results provide a direct assessment of costs and benefits of voluntary attention in a bird species. They show that crows are able to guide spatial attention using associative cues, and that the processing advantage at attended locations impairs performance at unattended locations.

## 1. Introduction

The attentional selection of environmental stimuli for enhanced processing at the expense of others is a common mechanism of the visual systems of many animal taxa [1,2]. Exogenous, stimulus-driven orienting of attention works in an automatic bottom-up direction and is a basic orienting mechanism across the animal kingdom [3,4]. By contrast, endogenous, voluntary attention is directed in a top-down manner and is independent from stimulus features [3]. Endogenous attention seems to be less universal in animals, as behavioural effects of voluntary orienting are primarily found in mammals and birds, but not yet in reptiles, amphibians or fish [1,5–10], with the potential exception of archer fish [11]. Invertebrates, especially visually foraging insects like bees, demonstrate stimulus competition and selection, and their spatially oriented behaviour is modulated by associative learning [12,13]. However, explicit tests of goal-directed, non-sensory selection in invertebrates are still needed. In mammals, behavioural effects of top-down attention allocation are associated with modulation of neural activity in visual cortical areas [14,15]. However, much less is known about how voluntary attention is mediated by the avian brain in the absence of a layered neocortex [16].

To investigate voluntary attention, psychophysical studies often use associative variations of the spatial Posner cueing task [17]. In this task, visual target stimuli typically must be detected or discriminated after the brief presentation of a cue stimulus. The information that the cue provides about the target determines the validity of the cue. A valid cue indicates the location of the upcoming target and can be used to direct spatial attention there before target onset. An invalid cue indicates a location opposite to the target, so it may misdirect attention. If most trials contain valid cues, animals may learn a predictive contingency between cue and target location and thus endogenously direct the attentional focus to the location associated with the cue. This results in improved performance such as faster reaction time (RT) or higher perceptual sensitivity (*d*′) for valid over invalid cue trials [16]. This validity effect for cues that are associated with a predictive relevance and displayed at a different location from the impending target is a behavioural indicator of endogenous attention [3].

Among birds, both barn owls [6] and chickens [7] show RT effects for central predictive cues. However, when pigeons were presented with peripheral cues at the possible target locations, they did not show a difference between exogenous, unpredictive cues and endogenous, predictive cues, suggesting they could not exploit predictive task information to guide behaviour [18]. A disadvantage of this study design is that using peripheral cues in the endogenous condition allows for stimulus-based visual priming. To dissociate voluntary orienting from stimulus-driven exogenous attention, it is important to present cue stimuli at a different location from where the peripheral targets appear. In a recent study in which we reported both an exogenous and an endogenous attention effect based on distinct time courses in crows, we had only used peripheral cues that appeared at the location of peripheral targets [10]. In the current study, we presented central informative cues on a front screen to reliably predict the location of peripheral targets presented on one of two opposite side screens, and thus at a different location from the cue.

Another aspect that could not be clarified from the previous study by Quest *et al*. [10] relates to the behavioural mechanism causing attentional cueing advantages in crows. It remains unclear whether the observed RT advantages in birds for valid over invalid cues result from a processing benefit for valid cues, a cost for invalid cues, or both. Costs and benefits of voluntary attention have been shown in humans by comparison of informative cues with a neutral control condition, e.g. for contrast sensitivity [19] and spatial acuity [20]. While the working definition of spatial attention includes both benefits at attended and costs at unattended locations [19], this cannot be assumed for valid-versus-invalid cueing effects without an assessment of the relative contributions of valid versus invalid cues to the overall effect. As hypothesized by Montagna *et al*. [20] for humans, endogenous cueing effects could also mainly result from benefits at attended locations with no or only reduced costs at unattended locations because endogenous attention is under flexible cognitive control [21]. This would mean that the invalidly cued attentional focus could be shifted fast enough to reduce initial behavioural costs. Although some animal studies of voluntary attention have included a neutral cue condition [22,23], a systematic trade-off of costs and benefits has not been investigated in birds so far. Moreover, although some studies, in addition to the valid cue condition, have included a no cue condition in which visual attention is not directed to any particular location before target onset [7,8,24,25], this too does not allow a reliable estimate of relative costs and benefits of spatial cueing. This is because presentation of the valid cue also provides non-spatial information about the task flow, allowing general arousal or motor response preparation to improve performance [26]. A behavioural advantage for valid cues over no cue trials in these studies may thus be driven in part by such spatially non-selective processes.

To account for such issues, comparison to a carefully chosen neutral cue stimulus would be better suited to reveal the relative magnitudes of both benefits and costs of spatial attention at varying cue–target delays in animals. We therefore used an endogenous Posner detection task with both valid, invalid and neutral associative cues. With this protocol, we show both RT costs and benefits of voluntary attention in two carrion crows.

## 2. Results

**Figure 1.**
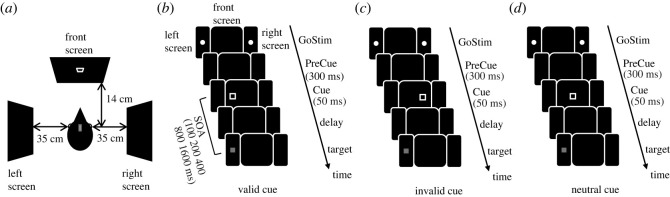
Experimental design of the endogenous attention task. (*a*) Set-up layout from top. The crows rested (with tracked head position) on a perch surrounded by two side screens for presenting the targets and a front screen showing the central cue. (*b*–*d*) In all task conditions, the crows had to detect a target after being visually cued. After a PreCue phase, a Cue stimulus (square outline) appeared on the front screen. Next, a variable Delay phase followed, defining the trial's SOA (100–1600 ms). Finally, the Target stimulus (grey square) appeared on either side screen, prompting the crows to respond. (*b*) Valid cue condition. In 70% of trials, the front cue was displaced 6.5 cm to the side of the impending target. (*c*) Invalid cue condition. In 10% of trials, the front cue was displaced 6.5 cm to the side opposite to the impending target. (*d*) Neutral cue condition. In 20% of trials, the front cue appeared in the screen centre and did not convey spatial information. Cue type, SOA and target side were presented pseudo-randomized.

We trained two carrion crows on an endogenous Posner spatial cueing task (figure 1). The crows had to detect a faint visual target on one of two opposite side screens after being spatially cued on a different front screen (figure 1*a*). In 70% of trials, the cue predicted target position and appeared 6.5 cm displaced to the side where the peripheral target would appear (valid cue, figure 1*b*). Thus, the spatial displacement of the cue indicated the future target side. In 10% of trials, the cue appeared 6.5 cm displaced to the side opposite to the upcoming target (invalid cue, figure 1*c*). The spatial cue thus had a validity of 87.5% and reliably indicated target side. In 20% of trials, the cue was centred on the screen, thus providing no spatial hint (neutral cue, figure 1*d*). Cue and target presentation were separated by a variable delay period. Cue and delay phase determined stimulus-onset asynchrony (SOA). We tested SOAs from 100 ms to 1600 ms.

While the spatially displaced cue was informative and highly valid regarding target side, the neutral cue was spatially uninformative. Therefore, both the validity effect of voluntary attention and its costs and benefits at attended versus unattended locations could be analysed by comparing detection accuracy and RT between the three cue types.

### 2.1. Costs and benefits of endogenous cueing

Both crows solved the task with high detection accuracy above chance, with an average 95.3% for crow 1 and 94.7% for crow 2 (both *n* = 25 sessions, all *p* < 10^−8^, binomial test, chance level 50%). To assess effects of endogenous cueing, a two-way ANOVA with cue type (valid, neutral, invalid) and SOA (100–1600 ms) as factors was performed for both RT and accuracy of each crow. The results are summarized in table 1. Both crows showed a significant main effect of cue type on RT (both *p* < 0.001, *n* = 25). Crow 2 additionally showed a significant RT effect of SOA (*p* < 0.001, *n* = 25). There was no interaction between cue type and SOA for either crow (both *p* > 0.05, *n* = 25). For detection accuracy, both crows showed a main effect of SOA (both *p* < 0.001, *n* = 25). No crow showed a main effect of cue type or an interaction between cue type and SOA (both *p* > 0.05, *n* = 25). Overall, cue type affected RT in both crows, while SOA but not cue type affected detection accuracy.

**Table 1.** ANOVA results. The results for both crows and both RT (top) and detection accuracy (bottom) are shown. *n* = 25 per crow, *α* = 0.05.

		crow 1				crow 2			
	factor	*SS*	d.f.	*F*	*p-*value	*SS*	d.f.	*F*	*p-*value
RT	cue type	16 165.3	2	15.8	2.62 × 10^−7^	29 384.6	2	42.2	3.39 × 10^−17^
	SOA	126.1	4	0.06	0.99	39 946.4	4	28.7	1.04 × 10^−20^
	cue type × SOA	1920.7	8	0.5	0.88	1757.1	8	0.6	0.75
accuracy	cue type	73.7	2	1.1	0.33	36.4	2	0.6	0.56
	SOA	1245.9	4	9.5	2.50 × 10^−7^	2216.6	4	17.4	4.33 × 10^−13^
	cue type × SOA	375.2	8	1.4	0.18	266.5	8	1.1	0.40

**Figure 2.**
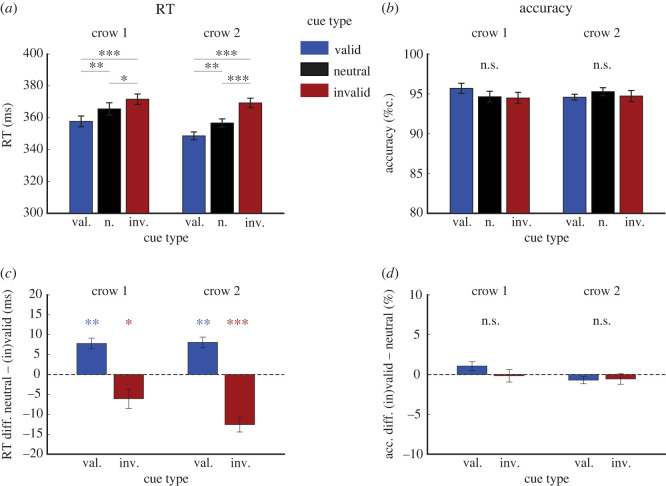
Costs and benefits of endogenous cueing. (*a*) Mean RT for all cue types across sessions for neutral (black), valid (blue), and invalid cues (red). (*b*) Mean detection accuracy for all cue types across sessions. (*c*) Average RT difference to the neutral cue average (dashed line) of valid (blue) and invalid (red) cue trials. (*d*) Average accuracy difference to the neutral cue average (dashed line) of valid (blue) and invalid (red) cue trials. All bars indicate the mean. Error bars indicate the standard error of the mean (s.e.m.), *n* = 25. Asterisks indicate significant pair-wise differences between cue types (*post hoc* Tukey's HSD test: *p < 0.05; **p < 0.01; ***p < 0.001; n.s.: not significant).

We analysed the effects of valid and invalid cues. Figure 2*a*,*b* shows the mean RT and detection accuracy for each cue type (all SOAs pooled). Average RTs were faster for valid cues (crow 1: 358 ms; crow 2: 349 ms) than for invalid cues (crow 1: 372 ms; crow 2: 369 ms) in both crows, resulting in a significant difference of 14 ms for crow 1 (*p* < 0.001, *n* = 25, *post hoc* Tukey's HSD test) and 21 ms for crow 2 (*p* < 0.001, *n* = 25, *post hoc* Tukey's HSD test). By contrast, detection accuracy did not differ between valid and invalid cues for either crow (both *p* > 0.05, *n* = 25, *post hoc* Tukey's HSD test). Therefore, there was a cue validity effect for RT but not accuracy across crows.

Next, we analysed the effects relative to neutral cues. Figure 2*c*,*d* shows the according costs and benefits of RT and accuracy for the valid and the invalid cue relative to the neutral cue average (baseline zero, dashed line). Positive values indicate benefits, i.e. shorter RT and higher accuracy compared to neutral cue trials. Negative values indicate costs, i.e. longer RT and lower accuracy compared to neutral cue trials. For valid compared to neutral cues, the crows showed RT benefits of 8 ms both (crow 1: *p* = 0.007; crow 2: *p* = 0.007; both *n* = 25, *post hoc* Tukey's HSD test). In addition, the crows showed RT costs for invalid compared to neutral cues of −6 ms (crow 1) and −13 ms (crow 2) (crow 1: *p* < 0.05; crow 2: *p* < 0.001; both *n* = 25, *post hoc* Tukey's HSD test). In contrast to RT effects, detection accuracy was unaffected for either cue type (both crows *p* > 0.05, *n* = 25, *post hoc* Tukey's HSD test). In summary, the crows showed both RT benefits for valid and costs for invalid cues, indicating that spatial miscuing affects the processing of task-relevant stimuli.

### 2.2. Temporal dynamics of endogenous cueing

**Figure 3.**
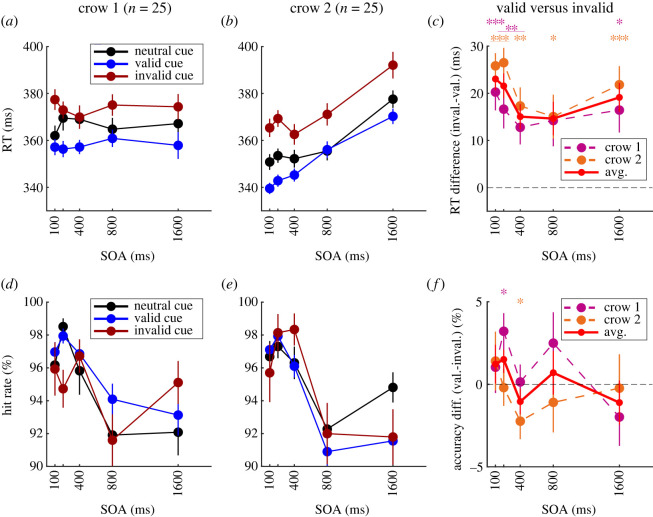
Temporal dynamic of cueing effects across SOAs. (*a*–*c*) RT data across SOAs. (*d*–*f*) Detection accuracy data across SOAs. (*a*) Mean RT for all cue types and SOAs of crow 1 and (*b*) of crow 2. (*c*) Mean RT difference between valid and invalid cue trials of crow 1 (magenta), crow 2 (orange) and the average between crows (red) at all SOAs. Positive values indicate faster RTs for valid cues than for invalid cues. The dashed line indicates the baseline, i.e. no difference between cue types. (*d*) Mean hit rate for all cue types and SOAs of crow 1 and (*e*) of crow 2. (*f*) Mean accuracy difference between valid and invalid cue trials. Conventions as in (*c*). Positive values indicate higher accuracy for valid than for invalid cues. All graphs indicate the mean, error bars indicate the s.e.m., *n* = 25. Asterisks indicate significant differences between valid and invalid cue trials at single SOAs within crows (Wilcoxon signed-rank test: *p < 0.05; **p < 0.01; ***p < 0.001).

To investigate the temporal dynamic of the observed cue validity effect, we compared RT between cue types for the different SOAs. We applied Bonferroni correction to RT and accuracy tests at all SOAs to account for multiple comparisons between valid, invalid and neutral cue data. Figure 3*a*,*b* shows the average RTs of both crows to the different cue types at all SOAs.

First, we compared valid to invalid cues. Endogenous spatial cueing led to a consistent validity effect over SOA. Both crows showed an RT advantage for valid over invalid cues of around 15–25 ms that was significant across SOAs (both crows *p* < 0.05, *n* = 25, Wilcoxon signed-rank test), except for crow 1 at 800 ms SOA (figure 3*c*). The full-blown validity effect was present already at 100 ms, the earliest SOA tested, in both crows with 20 ms and 26 ms difference (both *p* < 0.001, *n* = 25, Wilcoxon test). Thus, both crows showed a persistent and strong, positive RT effect, indicating that the valid cue improved detection speed for a spatially separate target across SOAs.

Next, we explored detection accuracy. Figure 3*d*,*e* shows that accuracy stayed above 90% across SOAs in both crows. There was no consistent advantage for valid over invalid cue trials for either crow (figure 3*f*): significant cueing effects were present only at 200 ms SOA for crow 1 (*p* = 0.027, *n* = 25, Wilcoxon test) and in the opposite direction at 400 ms SOA for crow 2 (*p* = 0.04, *n* = 25, Wilcoxon test). Overall, there were no SOA-specific validity effects of detection accuracy across crows.

Finally, we assessed costs and benefits of RT and accuracy for the different SOAs. RTs tended to be faster for valid and slower for invalid compared to neutral cues across SOAs by about 10 ms (electronic supplementary material, figure S1*a*,*b*). This difference was significant at most SOAs for crow 2 (*p* < 0.05, *n* = 25, Wilcoxon test), except for invalid cues at 400 ms and valid cues at 800 ms SOA. Crow 1 showed significant costs and benefits only at short SOAs in the range of 100–400 ms (*p* < 0.01, *n* = 25, Wilcoxon test), which may in part be due to the lower effect magnitudes compared to the overall validity effects. For detection accuracy, we did not find consistent costs or benefits across crows or SOAs (electronic supplementary material, figure S1*c*,*d*).

## 3. Discussion

We showed endogenous RT effects of central cues in two carrion crows consisting of both benefits at cued and costs at uncued locations. Cueing effects were stable across all tested SOAs. Our results complement previous findings by demonstrating that cueing effects of voluntary attention in crows are robust and can be long-lasting even when target stimuli are cued by central, associative stimuli. This finding corroborates and extends previous evidence for top-down spatial attention allocation in birds in the absence of a layered neocortex.

### 3.1. Costs and benefits of predictive cues

To compare the contributions of valid and invalid cues to endogenous RT effects, we presented neutral cues as a control condition in 20% of trials. The advantage of valid over invalid cues in our crows resulted from both RT benefits for valid and costs for invalid cue trials across SOAs. Therefore, the crows used the highly reliable spatial cue to allocate top-down attention to the cued side screen, and this came at a simultaneous processing cost for unattended locations. Overall, there was no benefit without a cost in either crow, indicating that voluntary attention is not easily disengaged in crows even after extensive training.

Most animal studies of cueing effects do not include a neutral cue presentation [5,6,11,18,27–29]. Several studies used a no-cue control instead, where the target appears without previous cueing [7,8,24,25]. However, a no-cue design does not account for the alerting effect of visual cues and may thus overestimate the effects of attention allocation [26]. Among birds, a study that does include neutral cues is the orientation discrimination task in chickens by Knudsen *et al*. [23]. They examined discrimination performance and perceptual sensitivity before and after focal lesions in the optic tectum of chickens using oriented Gabor gratings. When they compared a 100% valid cue with a neutral double cue presentation, they found behavioural benefits for the valid cue in two chickens before lesions, confirming that birds are able to use associative stimuli to guide voluntary attention. There was, however, no invalid cue condition, so it is unclear to what extent processing of unattended locations was impaired in these chickens. Moreover, the timing of cue and target presentation was self-paced in the task, so the relation between cue benefit and SOA is unclear. Sridharan *et al*. [7] tested chickens with 90% valid and 10% invalid cues at long SOAs above 1 s and found cueing effects, but they did not compare the invalid cue performance to a neutral condition. Our data thus contribute a direct assessment of the magnitude of endogenous attention costs in a bird species.

In overtly behaving rats, Bushnell [22] compared a 70% valid cue to a neutral double cue presentation at varying SOAs. In contrast to our results, they found both accuracy benefits for valid and costs for invalid cues across SOAs. In our task, accuracy was close to ceiling level in both crows across all conditions, so the task design might have concealed accuracy effects. Because our crows achieved high accuracy early on during initial training, this result is likely not an effect of simple overtraining. In fact, a similar cueing effect on accuracy emerged at lower target intensities in our previous, related protocol [10], so our target intensities may have been too far above perceptual threshold. Thus, without testing lower signal strengths, we cannot here draw conclusions about attentional accuracy trade-offs. Still, our results on RT support that the lasting cue validity effect is mediated by both benefits and costs even at extended SOAs. Taken together, the current results in birds and mammals point towards a common processing restriction for visual stimuli outside the focus of attention.

### 3.2. Central associative cueing

The presentation of frontal predictive cues facilitated or impaired the detection of a peripheral target in our crows, supporting that crows can spatially guide voluntary attention. We previously reported endogenous cueing effects in four crows using a task protocol with peripheral cue and target stimuli [10]. In that original task, overlying exogenous effects of cue presentation could not be dissociated from the endogenous condition because cue and target appeared at the same locations. In the current protocol, cues appeared spatially separate from the possible target positions, so the observed cueing effects across SOAs from 100 ms onward cannot be accounted for by stimulus-driven attention capture. Another explanation for the observed effects is a lateralized response bias to the cued visual hemifield resulting from the reward expectation associated with the spatial information the cue stimulus conveys [30]. This cannot be distinguished from a perceptual enhancement in the current task because our cue carried sufficient information to predict the precise location of the target. Importantly, the same issue would arise if we simply replaced our positional cues by a colour or arrow cue at a fixed central position. To resolve this confusion, the possible target locations can be orthogonalized to the spatial dimension of the cue, e.g. by varying the vertical in addition to the horizontal position. Such applications have differentiated between attentional enhancement and a separate response bias in chickens [7] and macaques [31]. Based on these results, we expect that crows also decode likely target location from the cue, actively disengage attention from the cue location and finally direct and maintain top-down attention at a cued target location, in addition to a potential response bias, but this requires further testing.

Our results further show that crows can benefit from informative cues as early as 100 ms, reflecting a faster time course than in humans [3]. The early onset of RT effects, their magnitude around 15–25 ms, and their maximum at short SOAs (100–200 ms) parallel the results in macaques for central predictive cues in a comparable Posner task [5], as well as in an overt saccade task (saccadic RT [31]). This suggests similar functional properties and constraints between corvids and primates despite very different brain architectures [32]. By contrast, RT effects of *peripheral* predictive cues in crows ramp up with longer SOAs and reach a maximum around 800 ms [10]. This pattern resembles sustained voluntary attention for central cues in humans [33], so the additional demand of decoding a spatially separated cue may impair attention maintenance in crows and macaques, whereas maintenance is preserved in humans.

Crows also show different effects for informative versus uninformative peripheral cues, i.e. when cue validity, and thus its predictive value, is changed [10]. Together with our results on central cues, the data from four crows suggest that they are sensitive to the value of a spatial cue with respect to current behavioural goals. When the cue is highly indicative of target position, the crows may learn to associate cue and target side to improve task-relevant performance, in this case detection speed. Crows have substantial working memory capacity [34–38] and are able to learn associations between abstract stimulus features even across modalities [39–41], enabling them to guide behaviour using associative stimuli. Similarly, barn owls show faster RTs to valid than to invalid visual cues with 80% validity in a sound localization task at SOAs of 500–1000 ms [6]. In an exogenous detection task, barn owls show a facilitation of RT at shorter SOAs and an inhibition at 600 ms SOA (‘inhibition of return’ [29]), suggesting an influence of cue validity on task performance. Pigeons, however, do not show distinct RT effects of cue validity when comparing exogenous and endogenous cueing [18]. It is unclear whether these diverging results reflect differential impacts of task design or true ecological differences across species.

## 4. Methods

### 4.1. Animals

Behavioural data were recorded in two carrion crows (*Corvus corone corone*), a 2.5-year-old male (crow 1) and a 1.5-year-old female (crow 2) born and raised at the university's facilities. The crows were kept in social groups in spacious aviaries. During training, crows were kept on a controlled feeding protocol, and food was used as reward. The crows always had *ad libitum* access to water. Both crows had previously been trained on a related attention task (crows 2 and 5 in [10]). All procedures were carried out according to the guidelines for animal experimentation and approved by the responsible authority under national legislation, the Regierungspräsidium Tübingen, Germany.

### 4.2. Experimental set-up

Training and data acquisition took place in a closed conditioning chamber (102 × 100 × 76 cm^3^) with a side door. The crows sat on a wooden perch 14 cm in front of a touch screen (3 M Microtouch, 15ʺ, 60 Hz refresh rate). On the side walls, two additional screens (Joy-it RB-LCD10-2, 10.1ʺ, 60 Hz refresh rate) were mounted at 35 cm viewing distance each. Figure 1*a* illustrates the configuration of front and side screens relative to the crow in the conditioning chamber, seen from the top. Below the front screen, the crows could retrieve food rewards from a custom-made automatic feeder. The task flow was controlled online using the CORTEX program (National Institute of Mental Health). Visual stimuli were presented on the front and side screens, auditory feedback was given by a speaker mounted behind the front screen. The crows' head position was tracked using a custom-written tracking program in Matlab. This program used the live feed of 2 infrared vision cameras (Body: FLIR CM3-U3-13y3M, Lens: Fujinon DF6HA-1B, IR-Emitter: Kingbright BL0106-15-28, 940 nm) in the chamber. One camera was mounted on the side wall left to the front screen and tracked the vertical plane, the other was mounted on the top wall and tracked the horizontal plane. The crows had to fixate head position throughout the task by keeping a small reflector attached on top of their head inside of a predetermined area centred between the two side screens. The crows were previously trained on this area by rewarding them for keeping their head position stable for an increasing amount of time. The allowed area for this reflector measured 2.7 cm × 2.7 cm × 2.5 cm, and the head angle had to remain within ± 20° of a straight forward orientation in the horizontal plane. Carrion crows have a wide frontal binocular field with a maximum width of about 38°, while the reported width for converged eyes in the plane containing the beak tip is 29° [42,43]. With our settings, the frontal cue stimuli were within the possible range of the crows' binocular field, whereas the peripheral target stimuli could only be seen monocularly. Cues and targets were thus projected onto different retinal areas, specifically the temporal versus the central retina. However, we do not think this influenced task performance because both areas have a relatively high ganglion cell density in jungle crows, similar to pigeons [44,45], so both stimuli could be processed with high visual resolution. To indicate detection of the target stimulus, the crows had to break fixation by moving their head outside of the tracking area, whereupon the feeder was briefly illuminated and delivered a reward.

### 4.3. Task protocol

The crows were trained on the Posner task for spatial selective attention [17], a well-established signal detection task used for testing humans as well as animal models. Behavioural data were recorded for a central-cue task with highly predictive cue stimuli. To assess the impact of both cue informativity and delay length on endogenous attentional effects, we measured detection accuracy and RT for a 3 × 5 factorial design with Cue Type (valid, invalid, neutral) and SOA (100, 200, 400, 800, 1600 ms) as factors.

The basic trial flow for the different cue types is shown in figure 1*b–d*. Trials started with an inter-trial interval (ITI) of 300 ms during which all screens remained dark. Next, a Go-stimulus in form of a white dot with a 4 mm diameter (0.7° viewing angle) on each of the side screens was presented for up to 30 s. During this time, the crow could initiate the trial by moving its head into the fixation position. In order to complete a trial, the crow had to stay in this position for all subsequent phases until the target appeared. When the crow entered fixation, the Go-stimulus disappeared, followed by a PreCue phase of 300 ms with all screens dark. Next, a spatial cue in form of a white square outline (2 × 2 cm^2^, 1 mm width, 2.95° viewing angle) was presented on the front screen for 50 ms. The position of the cue relative to the upcoming target determined a trial's cue validity. The cue was valid in 70% of all trials (figure 1*b*), invalid in 10% of all trials (figure 1*c*) and neutral in 20% of all trials (figure 1*d*). The proportion of valid to invalid cues corresponded to a cue validity of 87.5%. Valid and invalid cues were informative in that they conveyed correct or incorrect information about the location of the upcoming target, whereas neutral cues did not carry any information about the target location and served as baseline condition. A neutral cue was shown on the centre of the front screen. An informative cue (valid or invalid) was shown 6.5 cm displaced to the left or right of the screen centre. In valid cue trials, the cue was displaced towards the side screen where the target later appeared, whereas in invalid cue trials, it was displaced towards the opposite side. After cue offset, a delay phase of variable length (50–1550 ms) followed during which all screens remained dark. The length of the delay phase together with the length of cue presentation (50 ms) determined the trial's SOA. SOAs tested were 100, 200, 400, 800 and 1600 ms, presented pseudo-randomly across trials. After the delay, the target stimulus, a grey filled square (0.7 × 0.7 mm^2^, 1.15° viewing angle) with an intensity of 0.43 ± 0.02 cd m^−2^, appeared on one of the side screens at the position of the initial Go-stimulus. Target side was balanced across trials. Upon target presentation, the crow had to break fixation within 550 ms to make a correct response, meaning that they moved their head out of the fixation area via a ‘nodding’ gesture. If the crow did not respond in time, the trial was counted as an error and a timeout of 2000 ms with blank screens followed after a brief yellow flash. If it responded 150 ms or less after the target appeared, the trial was aborted to account for the processing latency in the visual pathway [46]. After the trial was aborted, a brief green flash was presented followed by a timeout of 2000 ms with blank screens.

An automated delayed retry protocol was used so that trials answered incorrectly would be drawn again later until all conditions were answered correctly once and a new block was initiated. After 60 correct trials, the crows had short breaks with access to water. The task went on until the crow did not initiate any more trials or until 2 h had passed.

### 4.4. Data analysis

Data acquisition took place once a day for each crow between 5 and 7 days a week. Twenty-five sessions were recorded per crow. Crow 1 completed between 300 and 540 correct trials per session (median = 480), crow 2 completed between 276 and 480 correct trials per session (median = 420). Behavioural task performance was measured as detection accuracy in % correct and RT in ms. Accuracy was computed by dividing the number of hits by the number of hits + misses. RT was measured for correct trials only from target onset within a response window of 150–550 ms. Aborted trials were not analysed. For each condition, mean accuracy and median RTs were computed per session. The distribution of session values was then summarized using the mean and standard error of the mean for both accuracy and RT.

To analyse detection accuracy and RT, two-way ANOVAs with Cue Type and SOA Length as factors were computed for each crow. Pairwise comparisons of cue types were performed *post hoc* using Tukey's HSD test.

To assess whether responses differed between cue types for a given SOA length, Wilcoxon signed-rank tests were performed using the individual session RT medians and performance means for each cue type and SOA. The respective central values were first subtracted from each other within sessions to obtain a measure of the RT and accuracy difference between cue types per session. Next, the differences of the individual sessions were tested against a median of zero (no effect of cue type). This way, both the advantage of valid cue trials over invalid cue trials and the costs and benefits of valid and invalid cue trials compared to neutral cue trials were analysed and Bonferroni-corrected for multiple comparisons.

All statistical tests had an alpha level of 5%. All statistical analyses and illustrations were performed in Matlab (MathWorks Inc., R2021b) using custom-written software.

## Data Availability

Data and code to reproduce all statistical analyses are published under the Open Science Framework and are available from the Dryad Digital Repository: https://doi.org/10.5061/dryad.tmpg4f53n [47]. The data are provided in electronic supplementary material [48].
